# Supplementary material for: Pharmacokinetics of oral moxidectin in individuals with Onchocerca volvulus infection
Source: PLoS Negl Trop Dis. 2022 Mar 25;16(3):e0010005. doi: 10.1371/journal.pntd.0010005 (PMC8986118; doi:10.1371/journal.pntd.0010005)
Supplement: Attachment — Submitted filename: MOXONC Phase 2 PK_Response_final.docx [file pntd.0010005.s001.docx]

| **Review Comments** | **Response** |
| --- | --- |
| **Reviewer 1** | |
| 1. The underlying hypothesis of this paper is that the pharmacokinetic profile of moxidectin would somehow be different in infected versus uninfected people.  Since moxidectin is not metabolized, it is difficult for me to see why this might be the case.  The authors should provide some additional rationale for undertaking the study.  Are there any other known incidence of infection affecting the kinetics of a non-metabolizable drug, especially in a chronic infection like onchocerciasis? | According to the drug label, the hepatic metabolism of moxidectin is minimal and renal elimination of intact drug is negligible (https://www.accessdata.fda.gov/drugsatfda_  docs/label/2018/210867lbl.pdf). It is known that much of the pathology of onchocerciasis is the direct consequence of localized host inflammatory responses to parasite antigens in the skin, eye and other tissues of the body (J Infect Dis., 1995 Mar, 171 (3): 659-671). Also, it is well known that infection and inflammation are associated with down-regulations of hepatic and extrahepatic cytochrome P450s, as well as other drug metabolizing enzymes and transporters (Clin Pharmacol Ther. 2009 Apr; 85(4): 434–438.), Drug Metabolism and Disposition 2020 Aug; 48 (8) 631-644). Thus, it is essential to explore potential PK changes for moxidectin in onchocerciasis patients to identify possible changes that might be overlooked in studies that only used healthy individuals to assesses drug PK, particularly where there may be important differences in healthy volunteer versus patient characteristics (e.g. weight, height, and body mass index) that may alter distribution. |
| 2. Another underlying hypothesis here is that the AUC of moxidectin in the infected individuals would be greater than ivermectin.  The authors did not address this at all.  Given they presumably had access to the matched cohort that was given ivermectin in the clinical trial, this is an unfortunate omission.  Adding these data would strengthen the manuscript considerably. | It was not an underlying hypothesis that the AUC of moxidectin would be different to ivermectin in infected individuals. The different PK of moxidectin compared to ivermectin was already well understood. The objective of the parental clinical trial (reference 18) was to determine in a small number of Onchocerca volvulus infected individuals whether moxidectin is safe enough to administer it in a future large study to further characterize moxidectin’s safety and efficacy. Blood samples were collected to describe the moxidectin in these individuals for the reason given in answer to question 1 above. However, there was no PK-related hypothesis in this trial as it was not designed for this purpose. The trial was not designed to compare moxidectin PK to ivermectin PK, and ivermectin concentration was not measured in the study. |
| 3. I originally had some serious concerns about the ethical underpinnings of the parental clinical trial.  The individuals were chosen for participation in the study based on not having had any treatment for onchocerciasis for five years prior to the start of the study.  As the clinical trial results were published in 2014 and Ghana reported treating all endemic villages as part of their elimination program by 1998, this seems to suggest the participants had been denied standard card leading up to the clinical trial.  However, in the paper referenced by the authors that refers to the parental clinical trial, it was clear that the villages from which the participants were drawn were hypo-endemic and outside the blinding onchocerciasis zone, and thus had not been enrolled in CDTI before the start of the clinical trial.  I think that this is very important information that needs to be included in this paper.  The authors need to expand on their process for enrollment, in order to allay these concerns. | We have modified the sentence “Participants were recruited from onchocerciasis endemic villages in the Tordzi basin in the Volta Region of South-eastern Ghana (details given in reference 18)” under section “Recruitment area and ethical approval” to include more information from reference 18: “Participants were recruited from villages in the Tordzi basin in the Volta Region of South-eastern Ghana.  The area was not included in vector control activities conducted by the Onchocerciasis Control Programme in West Africa because it was forested and not included in mass ivermectin distribution programmes because it was onchocerciasis hypoendemic (details given in reference 18).” |
| **Reviewer 2** | |
| 1. Line 107.  It is noted that moxidectin is more lipophilic than ivermectin – are the authors suggesting an advantage for moxidectin?  Please clarify the importance of including this statement | We’ve added the following highlighted text to clarify: “Moxidectin is more lipohilic (log P = 5.4) than ivermectin (log P = 4.3), resulting in a larger volume of distribution, greater uptake in adipose tissue, and partially explaining moxidectin’s longer half-life.” |
| 2. Line 183-184.  List the range for the standard curve. | The range for standard curve is added. |
| 3. Line 198.  Was Clast always above the LLOQ?  Were any concentrations below the LLOQ?  If so this should be identified and how handled discussed. | We’ve added text on how concentrations below LLOQ were handled under “Pharmacokinetic and statistical analysis.”: For the analysis, concentrations below lower limit of quantification were treated as missing. |
| 4. Line 200.  Both Cl and Volume of distribution should be labeled as “apparent”. | Corrections were made. Both Cl and Volume of distribution are labeled as “apparent”. |
| 5. Line 212.  The study referenced (#18) included 127 study participants who received moxidectin.  Why were only 98 included in this report? | It is correct that the parental study included 127 participants who received moxidectin. However, as detailed under section “Clinical study design and methodology”, Cohorts 3, 6 and 9 enrolled approximately twice as many participants as the mildly and moderately infected cohorts but blood samples for pharmacokinetics were obtained from only half of these. Hence the number of participants with pharmacokinetic data was 98. |
| 6. Line 216 – for the 2 mg dose 33 subjects were included (same in table1).  However in Figure 1 only 32 subjects are included – please clarify. | The number of subjects for 2 mg dose in Figure 1 was mislabeled. Figure 1 has been corrected to reflect n=33 for 2 mg dose. In addition, Figure 2 is also updated to correct the number of subjects for Cohort 1 (from n=9 to n=10). |
| 7. Table 2 – Tmax should be listed as median and range as it is considered a categorical variable and can only be either 3 or 4 as that is when sampling occurred. | Tmax values are now listed as median and range. Please note that as the analysis was conducted using actual sampling times (instead of nominal sampling times), Tmax values may not necessarily be exactly the same as planned sampling time. |
| 8. Table 3- Volume of distribution is highly variable, 3421 L for cohort 7 vs 1655 L for cohort 9.  This may warrant additional discussion, especially regarding demographics of cohorts (7,8,and 9). | The following text has been added to “Discussion”:  Cohort 7 was the only cohort with participants with measurable moxidectin concentrations at 6 and 12 months: one participant at 6 months, and one participant at 6 and 12 months. The very long elimination half-lives in these two participants resulted in high calculated parameters for V/F and thus a mean V/F for Cohort 7 much higher than the mean V/F for the other cohorts. No relationship with demographic characteristics could be established to account for this finding considering the relatively small number of participants in each cohort. |
| 9. The initial paragraph in the discussion really focuses on findings from reference 18.  I would suggest either moving to the introduction or rearranging the discussion to focus on findings of the current study. | Thanks for the suggestion. The initial paragraph in the discussion has been deleted, with part of the text moved to “Introduction”, and part of the text deleted altogether for more focus on findings of the current study, as suggested. |
| 10. Line 339 – not clear that ivermectin has a small apparent volume of distribution, perhaps smaller than moxidectin.  This should be clarified. | Line 339 (line 346 in the revised manuscript with racked changes) reports published data on ivermectin. We have re-introduced the reference [27] so that this is now clear. |
